# Detection of Antibodies against Tick-Borne Encephalitis Virus in Zoo Animals Using Non-Invasive Blood Sampling with Medicinal Leeches (*Hirudo medicinalis*)

**DOI:** 10.3390/pathogens10080952

**Published:** 2021-07-28

**Authors:** Pavel Kvapil, Marjan Kastelic, Nuša Jež, Kamil Sedlák, Nikola Kašpárková, Mateja Jelovšek, Tatjana Avšič-Županc, Eva Bártová, Jožko Račnik

**Affiliations:** 1 Ljubljana Zoo, Večna Pot 70, 1000 Ljubljana, Slovenia; marjan.kastelic@zoo.si (M.K.); nusa.jez1@gmail.com (N.J.); 2 Department of Biology and Wildlife Diseases, Faculty of Veterinary Hygiene and Ecology, University of Veterinary Sciences Brno, Palackého tř. 1946/1, 612 42 Brno, Czech Republic; kasparkova.nicola@seznam.cz (N.K.); bartovae@vfu.cz (E.B.); 3 Department of Virology and Serology, Prague State Veterinary Institute Prague, Sídlištní 136/24, 165 03 Prague, Czech Republic; kamil.sedlak@svupraha.cz; 4 Institute of Microbiology and Immunology, Faculty of Medicine, University of Ljubljana, Zaloška 4, 1000 Ljubljana, Slovenia; Mateja.Jelovsek@mf.uni-lj.si (M.J.); Tatjana.Avsic@mf.uni-lj.si (T.A.-Ž.); 5 Institute for Poultry, Birds, Small Mammals and Reptiles, Faculty of Veterinary Medicine, University of Ljubljana, Gerbičeva 60, 1000 Ljubljana, Slovenia; josko.racnik@vf.uni-lj.si

**Keywords:** infectious disease monitoring, animal welfare, epidemiology

## Abstract

Reports on non-invasive blood sampling are limited, and there are only a few studies on using kissing bugs (Reduviidae) and medicinal leeches (*Hirudo medicinalis*) for hematology and biochemistry testing in various zoo animal species. The aim of this study was to evaluate the usefulness of non-invasive blood sampling with medicinal leeches for arbovirus epidemiological investigations in various animal species from one zoo collection. Medicinal leeches were manually applied on 35 animals of 11 species. Control blood samples were obtained by venipuncture of the jugular vein. Antibodies to tick-borne encephalitic virus (TBEV) were detected by using the immunoenzymatic method or an immunofluorescent assay (IFAT), depending on the animal species. One of the 35 animals (2.9%) was seropositive (*Ovis aries*), whereas the rest of the samples were seronegative in both methods of sampling (non-invasive by leeches vs. invasive by venipuncture). Blood sampling using medicinal leeches showed promising results. It is likely a good alternative to other more complex and invasive methods, and it can provide significant advancement in blood sampling for preventive medicine and epidemiological studies in zoo animals.

## 1. Introduction

Animal welfare in zoos depends on good health care for each individual animal, with infectious disease monitoring being one of the most important pillars of preventive veterinary medicine in the collection. Blood samples are essential for hematological, biochemical, genetic, and endocrinological investigations, as well as for the screening of infectious diseases. However, blood sampling can be challenging because anesthesia is needed in most cases. The usefulness of bloodsucking bugs (family Reduviidae) for non-invasive animal blood sampling has already been described [1,2,3,4]; however, to the best of the authors’ knowledge, information on the usefulness of medicinal leeches (*Hirudo medicinalis*) from the family Hirudinidae for blood sampling has not been published yet. In the past, leeches were used for releasing bad humors in human and veterinary medicine [5,6]. Currently, they are used as additional therapy in plastic surgery and for treatment of osteoarthrosis and osteoarthritis [7,8,9,10]. Hirudin, excreted in leech saliva, is an anticoagulant agent secreted by the leech that prevents blood clotting and is also used in current human medicine [11]. Hirudin and several hirudin analogues have also been demonstrated to have several advantages as a highly specific anticoagulant over heparin, a widely used drug [12]. Moreover, hirudin is a potent thrombin inhibitor with similar characteristics to heparin, and so the difference between blood serum and plasma separated after centrifugation of hirudinized blood would likely mimic the differences between blood serum and blood plasma separated from heparinized blood. This study investigated the usefulness of medicinal leeches for blood sampling and serological screening of arboviral infection in various animal species in a zoo collection.

## 2. Brief Report

### 2.1. Animals

In total, 35 animals of 11 species (Table 1) from the zoo in Ljubljana, Slovenia, were included in this study. Medicinal leeches were obtained from a commercial source (Biopharm, Hendy, South Wales, UK) and were kept individually in sterile conditions in water at a temperature of 10 °C. One to three medium-sized starving leeches were manually applied to individual zoo animals. Venipuncture of the jugular vein was used to acquire control blood samples, collected at the same time in all the animals tested. The ambient temperature, humidity, and time taken to obtain the blood meal were measured from the beginning of the active sucking movement until the leeches spontaneously fell off the animals or were manually retrieved after achievement of sufficient enlargement. The individual leech was emptied, and blood volume was measured using a syringe with a varying volume (5, 10, or 20 mL) and a 22 G needle based on the size of the leeches.

### 2.2. Serological Methods

Two different serological methods were used to investigate the suitability of “hirudinized” blood obtained with medicinal leeches for the detection of antibodies against tick-borne encephalitis virus (TBEV). A total of 22 animals of four species (alpaca, goat, horse, and sheep) were investigated with the immunoenzymatic method following the manufacturer’s instructions (ELISA-EIA TBEV Ig, TestLine Clinical Diagnostic, Prague, Czech Republic). The diagnostic specificity was 95.7% and the diagnostic sensitivity was 95.7%.

All ELISA-positive samples were confirmed by a virus neutralization test (VNT) in micromodification, with vital staining. Briefly, a 100 μL working dilution of virus was mixed and incubated with an equal portion of the test serum and added to the same portion of the cell culture suspension. The plates were incubated in 5% CO_2_ at 37 °C. VNT was performed with TBEV (Strain Hypr) and concurrently with WNV (WNV Strain Line 2) and USUV (Austrian strain). Viruses were grown on the brains of suckling lab mice at 1–3 days of age. A suspension of a porcine kidney cell line (PS) was used as a cell substrate for TBEV VNT. A suspension of a monkey kidney cell line (CV-1) was used as a cell substrate for WNV VNT and USUV VNT. The working dilution for both cell lines was 600,000 cells/mL. Serum toxicity control was included in the tests, as well as specific positive and negative control sera. The result of VNT is a virus neutralization (VN) titer, which is the reciprocal of the highest sample dilution that is still capable of neutralizing the cytopathic effect at more than 50% (TCID50). Samples were marked as positive if the VN titer was >4. The virus neutralization test was performed in order to exclude other cross-reacting viruses (WNV, USUV) that may interfere with positive results.

In a further 13 animals of seven species (Alpine ibex, Bactrian camel, Chapman zebra, Kafue Flats lechwe, Red deer, Black swan, and Emu), the presence of antibodies to TBEV was investigated by indirect immunofluorescent assay (IFA) at the Institute for Microbiology in Ljubljana, Slovenia, using animal-species-specific anti-deer, anti-bovine, anti-horse, anti-sheep, and anti-bird conjugates (Bethyl Laboratories Inc., Montgomery, TX, USA). The sera were diluted to a ratio of 1:16 and tested using an indirect immunofluorescence assay on spot slides containing Vero E6 cells infected with TBEV (strain: Ljubljana I, U27494). In brief, the diluted serum was allocated on the slides. The slides were incubated for 30 min at room temperature and washed in phosphate-buffered saline (PBS), and afterward, anti-animal-species-specific IgG conjugate (Bethyl Laboratories Inc., Montgomery, TX, USA) diluted at the ratio of 1:128 was added. Another incubation at room temperature followed. Prior to examination under a fluorescent microscope (Eclipse 80i; Nikon, Melville, NY, USA), the slides were again washed in PBS and dried. The sera were considered positive when the characteristic TBEV cytoplasmic fluorescence was observed. The animal sera samples that were previously established as positive and PBS were used as positive and negative controls. The serum from seropositive sheep was further diluted to establish the end-point titer.

## 3. Results

Medicinal leeches were able to draw up to 20 mL of blood in about 8 to 55 min. External conditions such as light, temperature, and disturbing factors were crucial for the success of blood withdrawal as well as the time necessary for obtaining a sufficient volume of blood. The ambient temperature during sampling was between 7 and 23 °C, and humidity ranged from 38% to 70%. The success of blood withdrawal by medicinal leeches ranged from 33% to 100%. At least one out of the serological leeches applied drew blood. Only 1 (2.9%) of the 35 animals tested was seropositive for TBEV. Blood samples from a 6-year-old female sheep (*Ovis aries*) obtained by venipuncture as well as by leeches were positive using the ELISA method. Positivity for TBEV was confirmed by VNT in the sample from the vein with titer 64.

## 4. Discussion

The medicinal leech (*Hirudo medicinalis)* has been used for the treatment of many diseases for thousands of years. Bloodletting was a medical procedure believed to be a cure for anything from headache to gout [6]. Today, medicinal leeches are used for the treatment of osteoarthritis, venous congestion treatment in plastic and orthopedic surgery, and various purposes in alternative medicine [5,7,8,10,13]. Samples from wild-caught leeches have been used as a source of viral DNA and RNA of mammalian hosts [14]. To the best of the authors’ knowledge and based on an extensive literature review using the databases PubMed and Google Scholar, the use of medicinal leeches for non-invasive blood sampling and serological screening of infectious diseases has not been described.

Non-invasive methods of obtaining various samples in zoo and wild animals have been used in the past. Kissing bugs have been used as living syringes for obtaining blood samples in various animal species for analysis of hormone levels, genetic conditions, sexing, infectious disease monitoring, and hematological and biochemical parameters. The kissing bug, *Dipetalogaster maxima*, is the most common species used due to its large size and, therefore, large sample volume capacity [15,16]. Compared to blood-sucking bugs, leeches can obtain a larger blood meal and are able to survive and feed in a wider temperature range, between 0 and 30 °C. In our case, medicinal leeches were able to draw up to 20 mL of blood in about 8 to 55 min. However, sudden changes in environmental temperature can lead to stress, or even death of the leech [17]. Moreover, authors have observed that aside from temperature variance, smooth transport of leeches without shaking and trembling has a major impact on their willingness to move and suck.

In our study, three different serological methods (ELISA, IFAT, and VNT) were used to investigate the suitability of hirudinized blood obtained by medicinal leeches for detecting TBE antibodies in selected zoo animals. All the animals except one sheep tested negative. In the sheep, both the “leech” and control samples were positive. Due to the rapid redistribution of excessive fluid from the ingested blood, to secure the maximum capacity of the blood meal obtained by the leech, the plasma obtained would be expected to be present in sufficient quantities [11]. The antibodies tested in this study are part of the globulin protein fraction present in the blood. Consumption or digestion of these biological particles could be expected in the gut of the leech; however, results from previous biochemical studies of leech blood meals from various animals indicated that globulin values were never outside the physiological range [12]. Nonetheless, further investigation of blood-meal protein fractions and their durability/stability in the leech digestive tract could be beneficial [11].

Leech saliva also contains histamine-like substances that serve as a vasodilator in the area of sucking as well as hyaluronidase, which helps the leech penetrate the skin and better access the source of blood. Hirudin is also the most powerful natural anticoagulant known [11]. According to literature data, hirudin has the same antithrombotic action as heparin but with a much smaller dosage. This activity does not seem dose dependent, and it is associated with weak hemorrhagic effects [18]. In this context, one of the major disadvantages expected to impact the usefulness of medicinal leeches for blood sampling was extensive bleeding from the collection site due to the action of hirudin and other saliva contents after leech extraction. In human medicine, the bleeding time after leech removal is usually between 2 and 8 h [19]. In our cases, post-extraction bleeding was not remarkable. Unfortunately, it was not possible to evaluate the exact post-extraction bleeding time because most of the animals were released soon after blood sampling. However, in all 35 animals, no dripping or leaking of the blood from the wound was noticed 35 min after the leech removal.

A drawback of this study is the small pool of animals tested. A larger study with more positive samples could be beneficial for confirmation of the method described in this report. Blood sampling using medicinal leeches and their use for the detection of antibodies to arbovirus showed promising results. It is likely a good alternative to other more complex and invasive methods, and it can provide a significant advancement in blood sampling for preventive medicine and epidemiological studies in zoo animals.

## Figures and Tables

**Table 1:** Comparison of serological results obtained by leeches and conventional venipuncture.

No.	English Name	Latin Name	Age	Sex	Date of Sampling	TBEV Venous Serum (ELISA)	TBEV Serum from Leeches (ELISA)	TBEV/WNV/USUV, Venous Serum (VNT)	TBEV/WNV/USUV, Serum from Leeches (VNT)	TBEV/WNV/USUV/SINDBIS (IFAT)
1	Alpaca	*Vicugna pacos*	Adult	F	29 August 2017	neg.	neg.	neg.	neg.	ND
2	Alpaca	*Vicugna pacos*	Adult	F	29 August 2017	neg.	neg.	neg.	neg.	ND
3	Alpaca	*Vicugna pacos*	Adult	F	29 August 2017	neg.	neg.	neg.	neg.	ND
4	Alpaca	*Vicugna pacos*	Adult	F	1 September 2017	neg.	neg.	neg.	neg.	ND
5	Alpine ibex	*Capra ibex*	Adult	M	9 July 2018	ND	ND	ND	ND	neg.
6	Alpine ibex	*Capra ibex*	Adult	M	9 July 2018	ND	ND	ND	ND	neg.
7	Bactrian camel	*Camelus bactrianus*	Adult	M	30 August 2018	ND	ND	ND	ND	neg.
8	Black swan	*Cygnus atratus*	Adult	F	27 August 2018	ND	ND	ND	ND	neg.
9	Chapman zebra	*Equus quagga chapmani*	Juvenile	M	21 August 2018	ND	ND	ND	ND	neg.
10	Sheep	*Ovis aries*	Juvenile	M	30 August 2018	ND	ND	ND	ND	neg.
11	Emu	*Dromaius novaehollandiae*	Adult	F	11 December 2017	ND	ND	ND	ND	neg.
12	Emu	*Dromaius novaehollandiae*	Adult	M	11 December 2017	ND	ND	ND	ND	neg.
13	Goat	*Capra aegagrus hircus*	Adult	F	10 August 2017	neg.	neg.	neg.	neg.	ND
14	Goat	*Capra aegagrus hircus*	Adult	F	21 August 2017	neg.	neg.	neg.	neg.	ND
15	Goat	*Capra aegagrus hircus*	Adult	F	21 August 2017	neg.	neg.	neg.	neg.	ND
16	Goat	*Capra aegagrus hircus*	Juvenile	F	22 August 2017	neg.	neg.	neg.	neg.	ND
17	Goat	*Capra aegagrus hircus*	Adult	F	9 August 2017	neg.	neg.	neg.	neg.	ND
18	Goat	*Capra aegagrus hircus*	Adult	F	10 August 2017	neg.	neg.	neg.	neg.	ND
19	Goat	*Capra aegagrus hircus*	Juvenile	F	22 August 2017	neg.	neg.	neg.	neg.	ND
20	Horse	*Equus caballus*	Adult	M	21 August 2017	neg.	neg.	neg.	neg.	ND
21	Horse	*Equus caballus*	Adult	F	28 August 2017	neg.	neg.	neg.	neg.	ND
22	Horse	*Equus caballus*	Adult	F	28 August 2017	neg.	neg.	neg.	neg.	ND
23	Kafue Flats lechwe	*Kobus leche kafuensis*	Adult	F	16 July 2018	ND	ND	ND	ND	neg.
24	Red deer	*Cervus elaphus*	Adult	F	10 August 2018	ND	ND	ND	ND	neg.
25	Red deer	*Cervus elaphus*	Adult	F	10 August 2018	ND	ND	ND	ND	neg.
26	Red deer	*Cervus elaphus*	Juvenile	F	26 October 2018	ND	ND	ND	ND	neg.
27	Red deer	*Cervus elaphus*	Adult	M	26 October 2018	ND	ND	ND	ND	neg.
28	Sheep	*Ovis aries*	Adult	F	16 August 2017	neg.	neg.	neg.	neg.	ND
29	Sheep	*Ovis aries*	Adult	F	16 August 2017	neg.	neg.	neg.	neg.	ND
30	Sheep	*Ovis aries*	Adult	F	17 August 2017	neg.	neg.	neg.	neg.	ND
31	Sheep	*Ovis aries*	Adult	F	23 August 2017	neg.	neg.	neg.	neg.	ND
32	Sheep	*Ovis aries*	Adult	F	23 August 2017	neg.	neg.	neg.	neg.	ND
33	Sheep	*Ovis aries*	Adult	M	24 August 2017	neg.	neg.	neg.	neg.	ND
34	Sheep	*Ovis aries*	Adult	F	30 August 2017	neg.	neg.	neg.	neg.	ND
35	Sheep	*Ovis aries*	Adult	F	30 August 2017	positive	positive	64/neg/neg	TS	ND

TBEV—tick-borne encephalitis virus, WNV—West Nile virus, USUV—Usutu virus, VNT—virus neutralization test, SINDBIS—Sindbis virus, F—female, M—male, ND—not done, sheep no. 34 was seropositive for TBEV (titer 64), TS—toxic serum.

## Data Availability

The data that support the findings of this study are available on request from the corresponding author. The data are not publicly available due to privacy or ethical restrictions.
